# A Case of Recurrent Idiopathic Pyoderma Gangrenosum

**DOI:** 10.7759/cureus.25112

**Published:** 2022-05-18

**Authors:** Filipa David, Rafaela Lopes Freitas, Rute Brás-Cruz, Joana Rocha, Cristina Rosário

**Affiliations:** 1 Internal Medicine, Department of Medicine, Hospital Pedro Hispano, Matosinhos Local Health Unit, Matosinhos, PRT; 2 Dermatology, Department of Medicine, Hospital Pedro Hispano, Matosinhos Local Health Unit, Matosinhos, PRT

**Keywords:** immunosuppressive treatment, pathergy, necrotic ulcer, pyoderma gangrenosum, neutrophilic dermatosis

## Abstract

Pyoderma gangrenosum (PG) is a rare chronic neutrophilic dermatosis that can be associated with underlying conditions, such as inflammatory bowel disease and neoplasms, or can be idiopathic. Classically, it presents as painful skin lesions. We present a case of a 54-year-old woman who got a synovial cyst removed from her left hand, which later aggravated into a non-healing wound, and subsequently a painful necrotic ulcer. The histological pattern combined with the clinical features suggested PG. General wound care was performed, associated with topical tacrolimus and oral corticotherapy with a good response. Three similar episodes with lesions scattered over the body followed and required a combination of other pharmacological alternatives. An extensive etiological study was carried out to screen secondary causes without any relevant findings. Therefore, an idiopathic relapsing PG was assumed. PG is poorly understood, underdiagnosed and hard to treat. It has a clear impact on the quality of life of the patient, so high suspicion and timely treatment are essential to minimize complications.

## Introduction

Pyoderma gangrenosum (PG) is a rare, chronic and sometimes recurrent neutrophilic dermatosis, characterized by painful erythematous ulcerative lesions [1]. Neutrophilic dermatosis comprises a heterogeneous set of entities, to which Behçet's disease and Sweet's syndrome belong. However, despite having clinically distinct characteristics, histologically they present as an inflammatory infiltration with a predominance of neutrophils [2].

PG was first described in 1916 by Brocq, who called it “phagedenisme geometrique”, for its cutaneous presentation, and for some years it was believed that PG was related to disseminated streptococcal bacterial cutaneous infections with evolution to gangrene [3]. Nowadays, it is known that PG is a non-infectious inflammatory disease that affects about 3-10 per one million individuals, mostly females between the second and fifth decades of life [2]. The pathophysiological mechanism is complex and it is believed to be multifactorial, involving internal and external factors in the host [4].

The clinical presentation is diverse but there are five recognized types of presentation, which are based on the characteristics of the lesions and their behaviour: classic or ulcerative, bullous or vesiculobullous, vegetative, pustular and peristomal [2,3,5]. Ulcerative PG is the most frequent, corresponding to around 85% of identified cases, with small and painful erythematous or violaceous papules and pustules, which rapidly evolve to ulcers with an exudative, mucopurulent, haemorrhagic base or with areas of necrosis and high, well-defined, serpiginous and violet-blue or metallic grey borders, its distinctive feature, and they can be identified anywhere on the body surface [2,3,6]. These ulcerative lesions heal with shapeless and aberrant “cigarette paper-like” or cribriform scars [4].

## Case presentation

The case presented here is of a 54-year-old woman with uncomplicated arterial hypertension. In May 2018, she got a synovial cyst removed from the dorsum of the first metacarpophalangeal of her left hand. This became a difficult healing wound evolving in dehiscence of the surgical wound.

**Figure 1:**
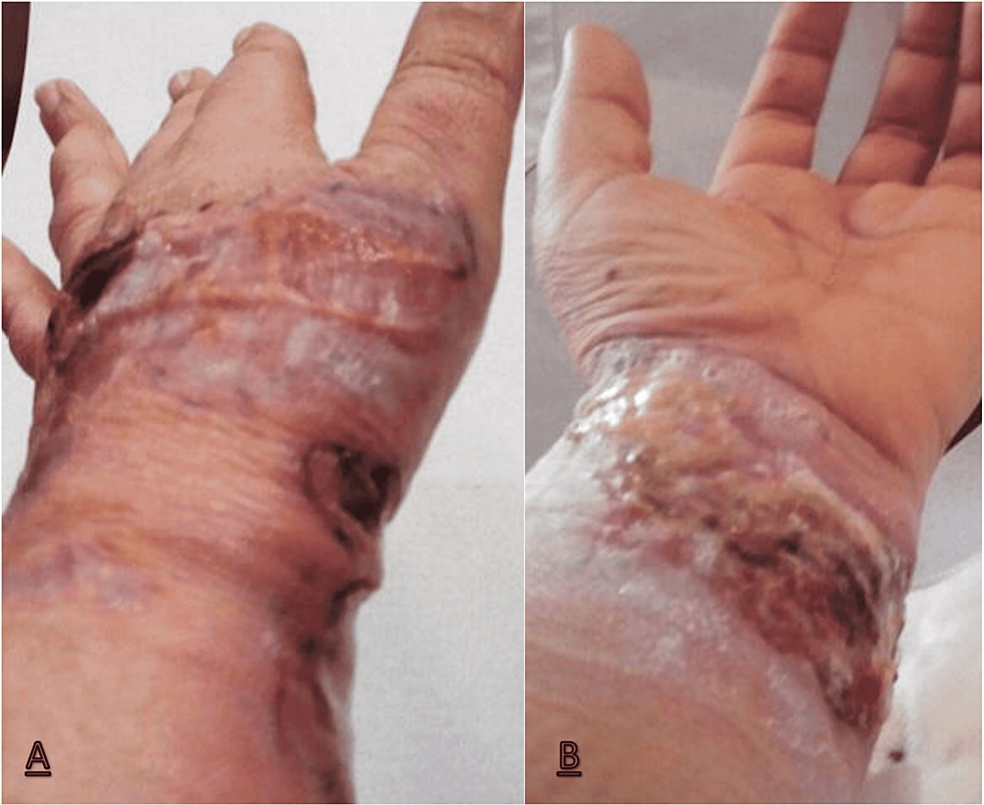
Painful necrotic ulcerated skin lesion on the left hand (A) and wrist (B)

A year later, there was a sudden appearance of a painful ulcerated lesion above the surgical scar that extended to the wrist (Figure 1). Then, it developed erythematous edges, central necrotic plaque and an intense foul odour. The histological pattern of the lesion, integrated with the clinical features, was compatible with PG. The patient underwent local treatment, with general wound care and topical tacrolimus, and systemic therapy with prednisolone 60 mg per day, with the resolution within three weeks.

**Figure 2:**
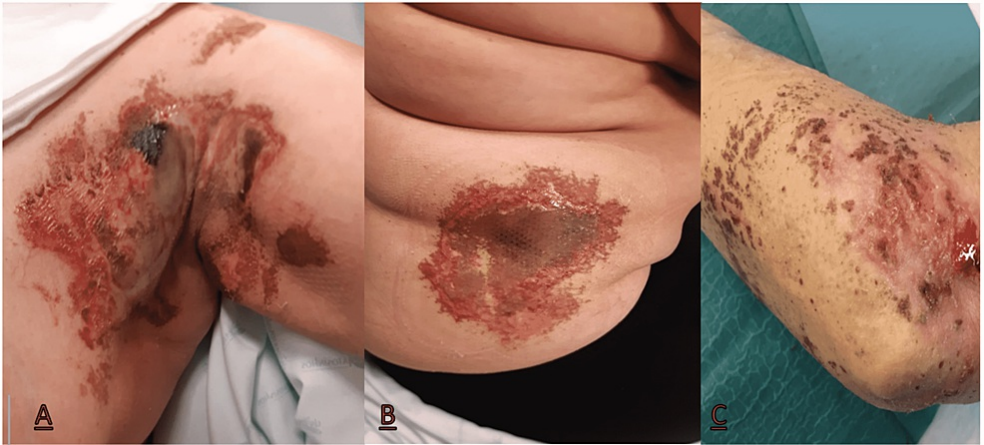
Painful necrotic ulcerated skin lesions of the medial wall of the left axilla and medial-axillary region of the chest wall (A), abdominal wall (B) and left forearm (C)

Similar episodes with lesions scattered over the trunk and upper limbs on the previously intact skin surface followed, in a total of three recurrences in three years (Figure 2). The first two recurrent episodes were treated as the inaugural event. However, given the recurrence of lesions and their appearance, gradually, in more areas of the body, cyclosporine 200 mg per day was added, with a good response, and a slow weaning from therapy was performed. In the last episode, due to a difficult arterial hypertension control, cyclosporine was replaced with dapsone (50 mg per day). The lesions completely healed in one month.

The etiological study was carried out. There was no history of recently introduced drugs, and we also excluded inflammatory bowel disease through endoscopic studies (upper and lower digestive endoscopies and capsule enteroscopy). In addition, paraneoplastic causes (screening for solid neoplasia through the skull and thoracic-abdominopelvic computed tomography, mammography and cervicovaginal cytology), autoimmune diseases (the immune study being unremarkable with no clinical clue supporting this entity) and haematological conditions (no analytical changes in peripheral blood immunophenotyping, serum protein electrophoresis and peripheral blood smears) were excluded.

## Discussion

PG is a challenging disease from diagnosis to treatment, and this is because it is uncommon, underdiagnosed and does not present any pathognomonic histological pattern or immunological markers [7-8]. Also, there aren't any duly validated diagnostic criteria or treatment guidelines. However, an effort has been made in an attempt to better understand the pathogenesis, because it may be the key to defining the best therapeutic strategies.

The pathophysiology of PG is complex and several mechanisms seem to interact, such as abnormality in the neutrophil function, dysregulation in the adaptative immune system by the imbalance between T cell subtypes (predominance of pro-inflammatory Th17 cells and a minority of regulatory T cells), and a dysfunction in the innate immune response with an expression and over-activation of multiprotein complexes, called inflammasomes, that promote an excessive release of pro-inflammatory cytokines [2].

As mentioned before, ulcerative PG is the most frequent clinical presentation and it was present in our patient. Regarding the symptoms caused by PG, pain is the most commonly reported symptom and also the most difficult to control, as in the case of our patient. However, patients may present with non-specific malaise, fever, arthralgia, or arthritis, as well as manifestations that reflect the involvement of internal organs, which may be related to other systemic diseases or just to the pathogenesis itself [3,7]. When assessing the present clinical case, it is clear that the first episode occurred in the same site where the initial surgical aggression took place - a phenomenon known as pathergy, where a small surgical intervention for minor trauma causes an exacerbated lesion when compared to the stimulus to which it was subjected. Pathergy occurs in around 20%-40% of PG cases, giving some sustainability to this diagnosis; however, it is not pathognomonic of the disease [3,5,6].

About 50% of PG cases are associated with systemic diseases (autoimmune, neoplastic, haematological) or secondary to drugs, and active investigation of these entities is mandatory. When there is no predisposing factor, as in our case, it is considered to be idiopathic [5,6]. The evolution of this disease is unpredictable, as well as its flares, and it can be indolent to extremely aggressive, with a very relevant impact on quality of life.

PG is a diagnosis of exclusion and it is consequently most often performed late. Recently, two diagnostic criteria have been proposed: Maverakis et al. created a Delphi consensus, which suggests that further investigation can be done only if we have a neutrophilic infiltrate histology, and Jockenhöfer et al. developed the PARACELSUS score based on the prevalence of several features typically found in PG [9,10]. Despite these efforts, early diagnosis is still difficult and, so far, no proposal has been validated.

Although there are no guidelines for the treatment approach, it is essentially based on reducing the inflammatory process, promoting healing and preventing infection, and depends on the severity of the clinical presentation. If mild, wound care and topical therapy with corticoids or immunosuppressants such as tacrolimus are recommended and, in moderate to severe forms, the choice is systemic, with steroids being the most effective method due to their potentially analgesic action and rapid resolution [6,11]. As alternatives, there are cyclosporine, dapsone, azathioprine, methotrexate and biologic agents [12]. Surgical debridement should be avoided due to the pathergy phenomenon [13]. In this case, the patient always developed new flares; hence, second-line drugs were needed in order, to not only treat but also prolong the time between exacerbations.

## Conclusions

PG is a rare disease with a tremendous impact on the quality of life, so it should be early diagnosed and actively excluded in patients where it is suspected. Although there are still no diagnostic criteria and protocol therapies, mostly due to its low incidence and difficult follow-up, a lot of research has been carried out in recent years to better understand the disease. Nevertheless, it is known that immunosuppression plays a major role in controlling the inflammatory response and using combination therapy improved its results. It is mandatory to search for underlying diseases and their management seems to be an asset for controlling and spacing the flares of cutaneous manifestations. Idiopathic PG treatment does not differ from other forms; however, the unpredictability of its manifestation may be greater. Finally, the authors reinforce the importance of managing these patients in multidisciplinary teams, as this disease has an impact on several biopsychosocial aspects. We emphasize the importance of this case for its rarity and the need to raise awareness for this entity in the medical community.
